# 750 Development of a Guideline for the Integration of Speech-Language Pathology into the Burn Multidisciplinary Team

**DOI:** 10.1093/jbcr/irad045.225

**Published:** 2023-05-15

**Authors:** Heather Cappel, Tiffany Mohr

**Affiliations:** Orlando Regional Medical Center - Orlando Health, Orlando, Florida; Orlando Regional Medical Center-Orlando Health, Sanford, Florida; Orlando Regional Medical Center - Orlando Health, Orlando, Florida; Orlando Regional Medical Center-Orlando Health, Sanford, Florida

## Abstract

**Introduction:**

Speech Language Pathology roles include assessment and treatment of communication, orofacial contractures and oral motor weaknesses, head and neck mobility, ventilator weaning, progression towards tracheostomy decannulation, and swallowing. Specific services may include augmentative and alternative communication, speaking valve implementation on and off the ventilator, advance instrumental assessment of swallowing, and innovative therapeutic interventions. The speech-language pathologists at a single verified burn center recognized a gap in knowledge regarding the scope services they provide resulting in underutilization by the multidisciplinary burn team. The purpose of this study was to develop and disseminate a proposed clinical guideline leading to the inclusion of the speech-language pathologist in the multidisciplinary burn team.

**Methods:**

Two hospital-based Speech-Language Pathologists undertook an exploratory research approach with an extensive review of available literature describing and defining their professional role both within the hospital system, and, more specifically, as a member of the burn multidisciplinary team. Burn team members were queried regarding their knowledge of speech services and appropriate patient referrals. Interviews and discussions with fellow professionals at other verified burn centers provided practice examples for comparison and consideration.

**Results:**

Results from the review of literature and interprofessional discussions within our institution and with other verified burn centers were synthesized into the development of a structured practice guideline. Speech-Language pathology was officially included in the burn multidisciplinary team at our institution. The guideline was shared with outside Speech-Language Pathologists who were not yet a part of their burn multidisciplinary teams to describe and validate the role.

**Conclusions:**

This foundational study led to increased awareness of Speech-Language pathology and the potential benefits to the hospitalized adult burn population. Active participation in the burn multidisciplinary conference has expanded patient care planning and led to generation of appropriate new consultations. Dissemination of the guideline supports the inclusion of Speech-Language in the burn multidisciplinary team.

**Applicability of Research to Practice:**

This guideline details the many specialty services provided by speech-language pathology and provides the foundation for future research to detail the impact on burn patient outcomes.

